# The association of the N-terminal pro-brain-type natriuretic peptide response to exercise with disease severity in therapy-naive pulmonary arterial hypertension: a cohort study

**DOI:** 10.1186/s12931-017-0712-9

**Published:** 2018-01-15

**Authors:** J. Kutsch, C. Faul, W. von Scheidt, M. Schwaiblmair, T. M. Berghaus

**Affiliations:** Department of Cardiology, Respiratory Medicine and Intensive Care, Klinikum Augsburg, Ludwig-Maximilians-University Munich, Stenglinstrasse 2, 86156 Augsburg, Germany

**Keywords:** N-terminal pro-brain-type natriuretic peptide (NT-proBNP), Response to exercise, Pulmonary arterial hypertension (PAH), Exercise capacity, Hemodynamics

## Abstract

**Background:**

While the N-terminal pro-brain-type natriuretic peptide (NT-proBNP) at rest is known to be associated with prognosis in pulmonary arterial hypertension (PAH), it is unclear if the NT-proBNP response to exercise (ΔNT-proBNP) can contribute to a better assessment of disease severity.

**Methods:**

We investigated the association of NT-proBNP values at rest and during peak exercise with hemodynamics and cardiopulmonary exercise testing parameters in 63 therapy-naive PAH patients.

**Results:**

The median NT-proBNP increases from 1414 at rest to 1500 pg/ml at peak exercise. The ΔNT-proBNP is baseline-dependent in PAH. Both, NT-proBNP at rest and NT-proBNP at peak exercise, are significantly correlated with hemodynamics and functional capacity. However, neither NT-proBNP at peak exercise nor ΔNT-proBNP correlated better with surrogate markers of disease severity than NT-proBNP at rest.

**Conclusion:**

The ΔNT-proBNP does not contribute to a better assessment of disease severity in PAH.

**Electronic supplementary material:**

The online version of this article (10.1186/s12931-017-0712-9) contains supplementary material, which is available to authorized users.

## Background

Although medical therapy is available, pulmonary arterial hypertension (PAH) is a progressive disease leading to a reduction in exercise capacity and, more severely, to a decreased life expectancy [1]. In order to evaluate the progress of the disease, a number of prognostic parameters were established, including hemodynamics and cardiopulmonary exercise testing (CPET) [2].

As right ventricular (RV) dysfunction is one of the most important factors contributing to functional impairment and mortality [3], the plasma concentration of N-terminal pro-brain-type natriuretic peptide (NT-proBNP) is supposed to be another prognostic marker. NT-proBNP is formed through enzymatic cleavage of proBNP into the two split products NT-proBNP and the biologically active BNP. ProBNP is released from the myocytes mainly in response to cardiac wall stress. BNP and NT-proBNP are produced on an equimolar basis, however, as NT-proBNP has a longer half-life and higher sample stability, NT-proBNP is the more advantageous diagnostic marker [4].

While the NT-proBNP level at rest is associated with prognosis in PAH [5], it is yet to evaluate if the NT-proBNP response to exercise (ΔNT-proBNP) can contribute to a better assessment of disease severity. Therefore, we investigated the association of NT-proBNP values at rest and during peak exercise with hemodynamics and functional capacity, important surrogate parameters of disease severity in PAH.

## Methods

### Study design

From August 2009 until March 2016, 63 therapy-naive PAH patients were recruited. Patients with a serum creatinine of >1.3 mg/dl, a glomerular filtration rate < 50 ml/min/1.73 m^2^, congenital heart diseases or signs of acute right heart decompensation were excluded from the study. All study participants underwent six-minute walking testing (6MWT), CPET, lung function testing and right heart catheterisation. Evaluation of exercise capacity and hemodynamics were used as surrogate parameters of disease severity in our trial. All examinations were performed within three consecutive workdays. The study was approved by the local Ethics Committee (project number 201604). Data analysis was performed retrospectively.

### Right heart catheterisation

In order to confirm precapillary pulmonary hypertension (PH), all patients underwent right heart catheterisation. A thermodilution catheter (7.5 F quadruple-lumen, balloon-tipped, flow-directed, „S “Tip Swan-Ganz Catheter, Edwards Lifesciences, Irvine, USA) was used. The catheter was inserted via the right or left femoral vein and hemodynamic measurements were taken in supine position. Those included heart rate, PCWP, PAP, cardiac output (CO), cardiac index (CI), pulmonary vascular resistance (PVR), right atrial pressure (RAP) and mixed venous oxygen saturation (SVO2). The CO was determined via thermodilution measurements. A computer system (Com-2, Cardiac Output Computer, Edwards Lifesciences, Irvine, USA) was used for calculations. 10 ml sterile, ice-cold isotonic (0.9%) saline was injected through the right atrial lumen of the catheter and measured by a thermistor placed directly behind the right atrial inlet of the catheter. The distal thermistor then recorded the drop in temperature. A minimum of three measurements was performed and the mean value was calculated provided that the variation was less than 10%. The CI was calculated forming the quotient of CO and body surface area. PVR was computed using the standard formula [PVR = (mean PAP-PCWP)/CO].

### Lung function test

In order to examine the pulmonary function of the patients, spirometry and body plethysmography were performed. The diffusing capacity was tested via the single-breath method (Master Screen Body and MS-PFT, Jaeger, Cardinal Health, USA). Blood gas analysis was measured using arterialised capillary blood taken from the earlobe. No supplemental oxygen was given. Lung function parameters determined were total lung capacity (TLC), forced vital capacity (FVC), forced expiratory volume in one second (FEV1) and diffusing capacity for carbon monoxide (DLCO).

### Cardiopulmonary exercise testing (CPET)

An electromagnetically braked cycle ergometer (ViaSpring 150p, Ergoline, Germany) was used following a standardised protocol [6].

After a warming phase, the work rate elevated by 5 to 15 watts/min until the maximum exercise capacity was reached. This was defined as the moment when patients could no longer tolerate the symptoms. Maximum work rate was documented. While the pulse was continuously recorded, the blood pressure was measured non-invasively every two minutes. With an adult facemask (Vmax spectra 229 D, Sensor Medics, USA) minute ventilation (Ve) and CO_2_ output were recorded. The maximum O_2_ uptake (peak VO2) was determined taking the average O_2_ uptake during the last 15 s of CPET. Blood gas analysis was done at rest and at peak exercise. We calculated O_2_ pulse, alveolar-arterial O_2_ difference (AaDO2) and functional dead space ventilation (Vd/Vt). The anaerobic threshold (AT) was defined as the O_2_ uptake patients reached in the moment the ventilatory equivalent for O_2_ (VE/VO2) still increased and the ventilatory equivalent for CO_2_ (Ve/CO_2_) decreased or stagnated. The VE/VCO2 slope was taken from the beginning of exercise until the respiratory compensation point, when acidaemia stimulated the ventilation and the end-tidal CO_2_ started to decrease.

### NT-proBNP

NT-proBNP values were measured twice using capillary blood taken from the earlobe immediately before performing the CPET and after reaching peak exercise capacity. The one-step sandwich chemiluminescent immunoassay (Dimension Vista System, Siemens Healthcare Diagnostics Inc., Newark, USA) was used. In order to avoid an underestimation or overestimation of absolute values, measured NT-proBNP levels were divided by the age-adjusted normal upper range to calculate the normalised NT-proBNP ratio. Consequently, elevated levels result in a normalized NT-proBNP ratio > 1.

### Six-minute walking test (6MWT)

Patients were encouraged to walk a 30 m long corridor as many times as possible in six minutes. The number of breaks was recorded. The achieved walking distance was set in ratio to age and gender. Immediately after the test, patients were asked to rate their perceived level of exhaustion on the Borg scale ranging from 0 (no dyspnea) to 10 (extreme dyspnea) [7].

### Statistics

Statistical analysis was performed with IBM SPSS Statistics 23. Data was checked for normal distribution using graphic methods as well as the Shapiro-Wilk test. Mean and standard error of the mean (SEM) were calculated for data with normal distribution, median with range for those without.

Nominal variables are shown as numbers with percentage of total. We used the Spearman’s rank correlation coefficient to examine the relation between NT-proBNP values and other parameters. Correlation is strongest if the coefficient is close to −1 or 1. No correlation is apparent if the coefficient is 0. Significance was tested two-tailed and assumed statistically significant if *p*-values were <0.05.

## Results

### Patients’ characteristics

Sixty-three patients (32 females and 31 males, mean age 66.1 ± 1.7 years) could be included in our study. All study participants suffered from PAH with a mean PAP of 39.0 ± 1.5 mmHg. The median NT-proBNP value at rest was 1414 (38–13,538) pg/ml. In Table 1, further clinical characteristics of the study cohort are summarised.

**Table 1:** Patients characteristics (*n* = 63)

Clinical profile	
Female/male [n (%)]	32 (50.2) / 31 (49.8)
Age (years)	66.1 ± 1.7
BMI (kg/m^2^)	26.0 (18.0–43.0)
NT-proBNP	
NT-proBNP at rest (pg/ml)	1414 (38–13,538)
NT-proBNP at exercise (pg/ml)	1500 (42–14,365)
NT-proBNP ratio	6.95 (0.09–60.60)
ΔNT-proBNP (pg/ml)	77 (0–1160)
6-min walking test	
Distance (m)	300 (100–570)
% of norm (%)	63.0 ± 3.2
Breaks (n)	0 (0–4)
Borg scale points (1–10)	4 (0–10)
Right heart catheterisation	
Mean PAP (mmHg)	39.0 ± 1.5
Cardiac output (l/min)	4.10 (2.30–8.90)
Cardiac index (l/min/m^2^)	2.35 ± 0.10
PVR (Wood Units)	7.45 (3.04–14.20)
Mean RAP (mmHg)	6.00 (1.00–20.00)
SvO_2_ (%)	61.1 ± 1.04
Lung function	
DLCO (%)	52.6 ± 3.30
PaO_2_ at rest (mmHg)	58.0 ± 1.70
PaO_2_ at peak exercise (mmHg)	58.0 ± 2.27
Cardiopulmonary exercise testing	
Work capacity (watts)	45.0 (25–122)
VO_2_ (ml/min)	941 ± 41.5
VO_2_ (ml/min/kg)	12.7 ± 0.5
AT (ml/min/kg)	9.43 ± 0.37
O_2_ pulse at peak exercise (ml/min/beat)	8.46 ± 0.34
Ve (L/min)	50.8 ± 2.23
Ve/VO_2_	39.0 (25.0–77.0)
Ve/VCO_2_	44.0 (28.0–87.0)
AaDO_2_ (mmHg)	48.1 ± 2.2
Vd/Vt (%)	36.7 ± 1.7
Ve/VCO_2_ Slope	39.5 (14.3–107)

When not stated otherwise, data are presented as mean ± SEM or as median (range)

### NT-proBNP levels in different risk groups

Based on the NT-proBNP level at rest, the study population was divided into three groups. The NT-proBNP <300 pg/ml group showed a median response to exercise of 6 pg/ml, the NT-proBNP 300–1400 pg/ml and the NT-proBNP >1400 pg/ml groups showed higher absolute ΔNT-proBNP, 60 pg/ml and 200 pg/ml, respectively. Additional information is shown in Table 2.

**Table 2:** NT-proBNP response to exercise according to different NT-proBNP levels at rest

NT-proBNP at rest	<300 pg/ml		300–1400 pg/ml		>1400 pg/ml	
Number (n)	10		21		32	
NT-proBNP at rest	84	(38–246)	821	(426–1300)	3145	(1414–13,538)
NT-proBNP at exercise	95	(42–281)	844	(450–1500)	3251	(1500–14,365)
∆NT-proBNP absolute	10	(0–49)	60	(0–400)	200	(0–1160)

When not stated otherwise, data are presented as median (minimum - maximum) in pg/ml

### Correlations of NT-proBNP and ΔNT-proBNP with hemodynamics and exercise capacity

Strong and highly significant correlations were found between NT-proBNP at rest and at peak exercise with parameters of 6MWT, hemodynamics and CPET. With the majority of parameters, NT-proBNP at rest showed marginally better correlations than NT-proBNP at exercise. Exceptions are CO and PVR. In contrast, lung function parameters showed only weak correlations with NT-proBNP levels at rest or at peak exercise. The ΔNT-proBNP only correlated significantly with the 6MWT distance, AaDO_2_ and the Ve/VCO_2_ slope (see Table 3). Correlations of NT-proBNP and ΔNT-proBNP with the 6MWT distance, mean PAP and the VO_2_/kg are shown as plots in Additional file 1. They show that the correlations of ΔNT-proBNP values with hemodynamics and functional capacity are worse compared to NT-proBNP levels at rest or at peak exercise.

**Table 3:** Correlation of NT-proBNP and ∆ NT-proBNP with hemodynamics and exercise capacity at rest and at peak exercise

		NT-proBNP at rest				NT-proBNP at peak exercise		∆ NT-proBNP	
		level		ratio		level		level	
	n	r	p	r	p	r	p	r	p
6MWT									
Distance (m)	61	−0.507	<0.001	−0.328	0.010	−0.490	<0.001	−0.256	0.047
% of norm	61	−0.546	<0.001	−0.481	<0.001	−0.534	<0.001	−0.310	0.015
Breaks (n)	63	0.556	<0.001	0.484	<0.001	0.541	<0.001	0.183	0.152
Borg scale	63	0.271	0.032	0.245	0.053	0.260	0.039	0.033	0.800
Hemodynamics									
Mean PAP (mmHg)	63	0.341	0.006	0.521	0.001	0.335	0.007	0.110	0.391
Cardiac output (l/min)	62	−0.322	0.011	−0.228	0.074	−0.325	0.01	−0.215	0.093
Cardiac index (l/min/m^2^)	36	−0.384	0.021	−0.360	0.031	−0.384	0.021	−0.248	0.145
PVR (wood units)	56	0.359	0.007	0.476	<0.001	0.361	0.006	0.226	0.094
Mean RAP (mmHg)	57	0.231	0.083	0.281	0.034	0.220	0.100	0.149	0.269
SVO_2_ (%)	61	−0.530	<0.001	−0.443	<0.001	−0.515	<0.001	−0.208	0.107
Lung function									
DLCO (%)	44	−0.332	0.028	−0.270	0.077	−0.314	0.038	−0.182	0.238
PaO_2_ at rest (mmHg)	63	−0.312	0.013	−0.233	0.066	−0.295	0.019	−0.029	0.824
PaO_2_ at exercise (mmHg)	63	−0.221	0.081	−0.174	0.171	−0.202	0.113	−0.068	0.599
CPET									
Work (watts)	63	−0.438	<0.001	−0.280	0.026	−0.410	0.001	−0.074	0.567
VO_2_ (ml/min)	63	−0.492	<0.001	−0.335	0.007	−0.473	<0.001	−0.136	0.289
VO_2_/kg	63	−0.514	<0.001	−0.436	<0.001	−0.490	<0.001	−0.193	0.130
AT (ml/min/kg)	48	−0.307	0.034	−0.335	0.062	−0.272	0.020	−0.165	0.263
O_2_ pulse (ml/min/beat)	62	−0.362	0.004	−0.290	0.022	−0.356	0.004	−0.089	0.491
Ve (l/min)	63	−0.024	0.853	0.092	0.472	−0.007	0.955	0.183	0.151
Ve/VO_2_	49	0.479	<0.001	0.398	0.005	0.463	0.001	0.241	0.096
Ve/VCO_2_	49	0.465	0.001	0.361	0.011	0.446	0.001	0.219	0.131
AaDO_2_ (mmHg)	63	0.452	<0.001	0.395	0.001	0.442	<0.001	0.320	0.011
Vd/Vt (%)	62	0.349	0.005	0.296	0.019	0.335	0.008	0.130	0.314
Ve/VCO_2_ slope	62	0.471	<0.001	0.435	<0.001	0.466	<0.001	0.330	0.009

## Discussion

Our study was conducted in order to evaluate if the NT-proBNP response to exercise can contribute to a better assessment of disease severity in PAH.

First, we were able to demonstrate that NT-proBNP levels increase in response to exercise in PAH patients. Similar findings have already been made in studies investigating left heart disease [8–10], and in one very small cohort, consisting of only 20 patients with precapillary PH [11]. However, there is another trial addressing the ΔNT-proBNP in PAH patients which showed no such effect [12]. While the design of the study was comparable to ours, patients investigated by *Völkers* and colleagues [12] were already on specific PAH medication and showed much lower NT-proBNP levels at rest. In left heart disease, low NT-proBNP levels at rest are supposed to be strong predictors for low exercise-induced changes [9, 10]. Thus, we divided our patients into three groups, based on the NT-proBNP levels at rest. The groups were formed according to the current NT-proBNP-based risk stratification model for clinical worsening or short-term mortality in PAH [1]. While only a very mild ΔNT-proBNP could be demonstrated for the group with a low NT-proBNP at rest, the NT-proBNP response to exercise increased with rising baseline levels. For example, comparable exercise-induced NT-proBNP changes could be demonstrated in patients from our low-level group and study participants in the trial by *Völkers* et al. [12]. We therefore conclude that, like in left heart disease [9, 10], NT-proBNP at rest seems to be a predictor for the exercise-induced ΔNT-proBNP.

PH patients with a strong NT-proBNP response to exercise are supposed to be limited primarily by RV dysfunction due to an impaired lung perfusion. Indeed, we found in PAH that both NT-proBNP at rest and NT-proBNP at peak exercise are significantly correlated with hemodynamics. These findings are in accordance with an earlier study, where NT-proBNP at rest has been found to be an independent predictor for hemodynamic parameters in cardiopulmonary diseases [3]. We also found a strong correlation of NT-proBNP and NT-proBNP at peak exercise with CPET parameters, as exercise capacity is believed to be primarily limited by cardiopulmonary function [13]. No such correlations were found between NT-proBNP and its response to exercise with parameters of lung function, as NT-proBNP gives only indirect information about ventilation. Because the NT-proBNP response to exercise is baseline-dependent in PAH, neither NT-proBNP at peak exercise nor ΔNT-proBNP correlated better with prognostic parameters than NT-proBNP at rest.

Unarguably, our study has limitations. First, due to the advanced age of the study cohort, comorbidity might have biased the results. To reduce this effect, we excluded patients with severe kidney dysfunction, congenital heart disease or patients with acute right heart failure. Second, the NT-proBNP response to exercise might be different in PAH subgroups. Finally, the prognostic relevance of our observations remains unclear, as our study does not include long-term follow up results.

## Conclusions

Despite these limitations, we conclude the following: NT-proBNP increases in response to exercise in PAH. The ΔNT-proBNP seems to be baseline-dependent. Both NT-proBNP at rest and NT-proBNP at peak exercise are significantly correlated with hemodynamics and functional capacity. Neither NT-proBNP at peak exercise nor ΔNT-proBNP correlated better with surrogate markers of disease severity than NT-proBNP at rest. Thus, the NT-proBNP response to exercise does not contribute to a better assessment of disease severity in PAH.

## Additional file

Additional file 1: Correlation plots of NT-proBNP at rest and at peak exercise and the ΔNT-proBNP with the 6MWT distance, mean PAP and the VO_2_/kg. (ZIP 540 kb)

## Abbreviations

6MWT: Six-minute walking testing
AaDO_2_: Alveolar-arterial oxygen difference at peak exercise
AT: Anaerobic threshold
BMI: Body mass index
CI: Cardiac index
CO: Cardiac output
CPET: Cardiopulmonary exercise testing
DLCO: Lung diffusing capacity for carbon monoxide
FEV1: Forced expiratory volume in one second
FVC: Forced vital capacity
NT-proBNP ratio: NT-proBNP level at rest divided by the age-adjusted normal upper range
NT-proBNP: N-terminal pro-brain-type natriuretic peptide
PAH: Pulmonary arterial hypertension
PaO_2_: Arterial oxygen pressure
PAP: Pulmonary arterial pressure
PCWP: Pulmonary capillary wedge pressure
PH: Pulmonary hypertension
PVR: Pulmonary vascular resistance
RAP: Right atrial pressure
RV: Right ventricular
SEM: Standard error of mean
SvO_2_: Mixed venous oxygen saturation
TLC: Total lung capacity
Vd/Vt: Functional dead space ventilation at peak exercise
Ve: Peak minute ventilation
Ve/VCO_2_ slope: Slope of minute ventilation to carbon dioxide output
Ve/VCO_2_: Carbon dioxide equivalent at anaerobic threshold
Ve/VO_2_: Oxygen equivalent at anaerobic threshold
VO_2_: Peak oxygen uptake
ΔNT-proBNP: NT-proBNP level at exercise minus NT-proBNP level at rest
