# Supplementary figures and images for: The association of the N-terminal pro-brain-type natriuretic peptide response to exercise with disease severity in therapy-naive pulmonary arterial hypertension: a cohort study
Source: Respir Res. 2018 Jan 15;19:8. doi: 10.1186/s12931-017-0712-9 (PMC5769493; doi:10.1186/s12931-017-0712-9)

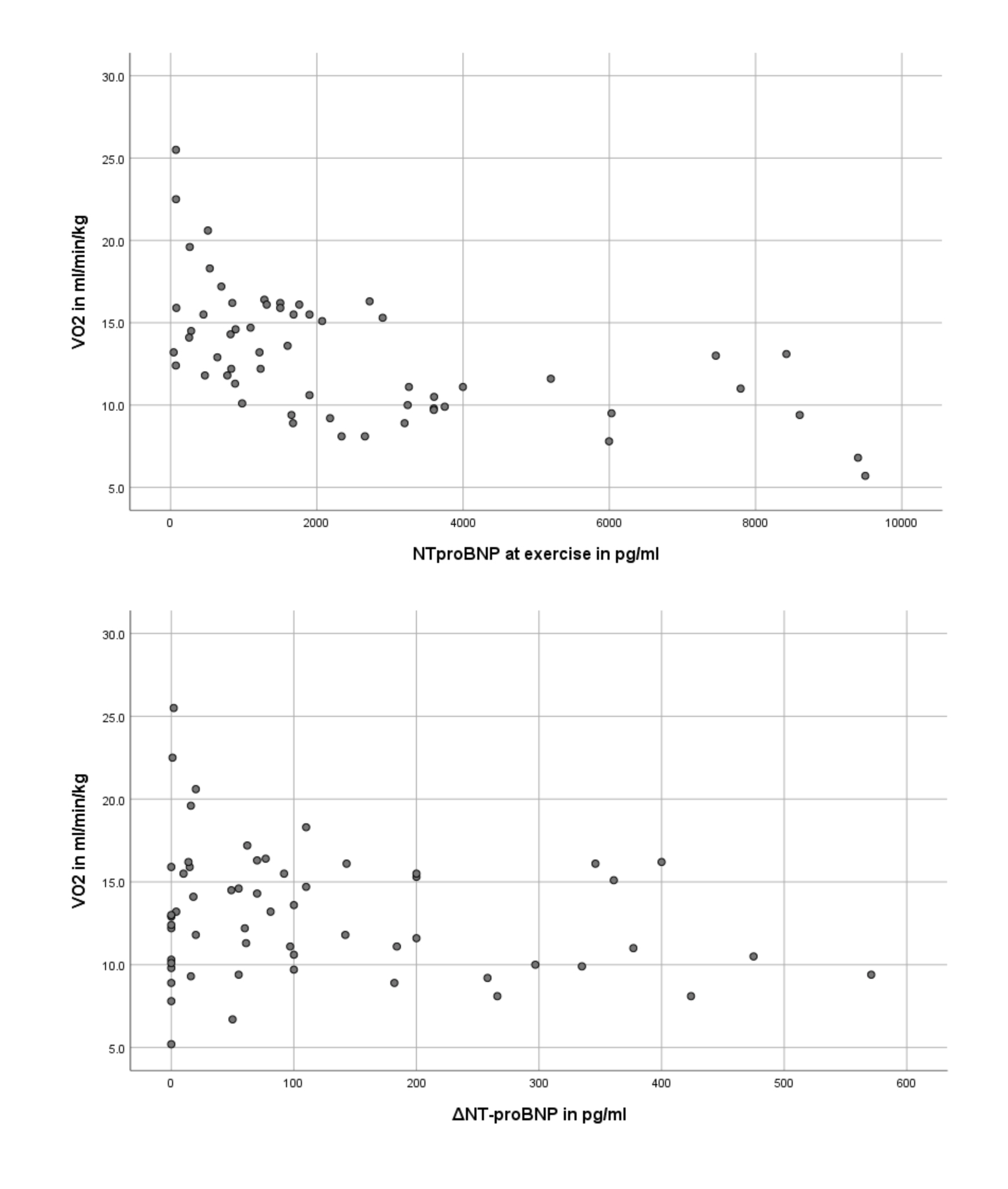

Supplement: Additional file 1: — Correlation plots of NT-proBNP at rest and at peak exercise and the ΔNT-proBNP with the 6MWT distance, mean PAP and the VO2/kg. (ZIP 540 kb) [file 12931_2017_712_MOESM1_ESM.zip › RERE-D-17-00298-R2-Figure Part 4.jpg]

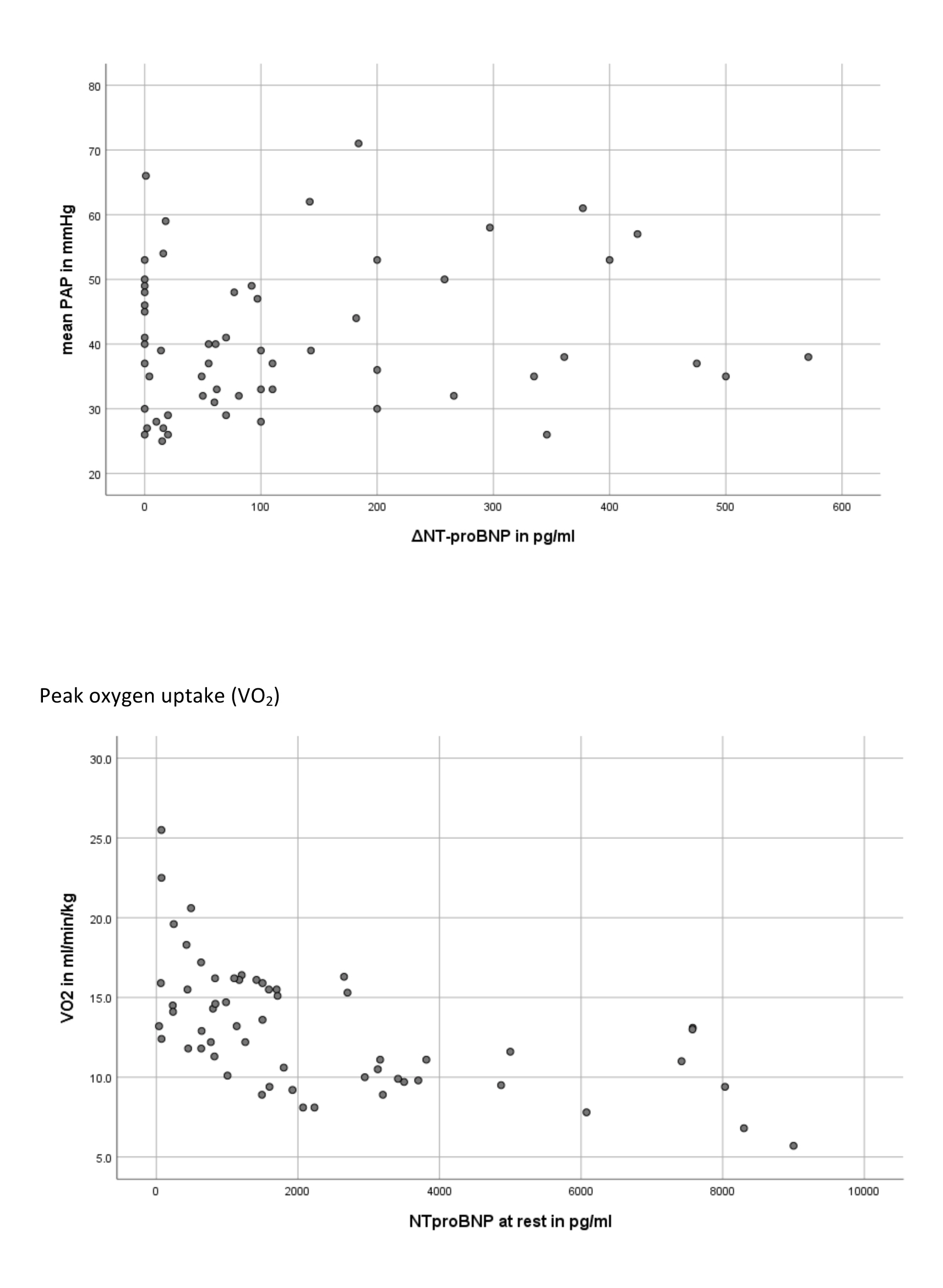

Supplement: Additional file 1: — Correlation plots of NT-proBNP at rest and at peak exercise and the ΔNT-proBNP with the 6MWT distance, mean PAP and the VO2/kg. (ZIP 540 kb) [file 12931_2017_712_MOESM1_ESM.zip › RERE-D-17-00298-R2-Figure Part 3.jpg]

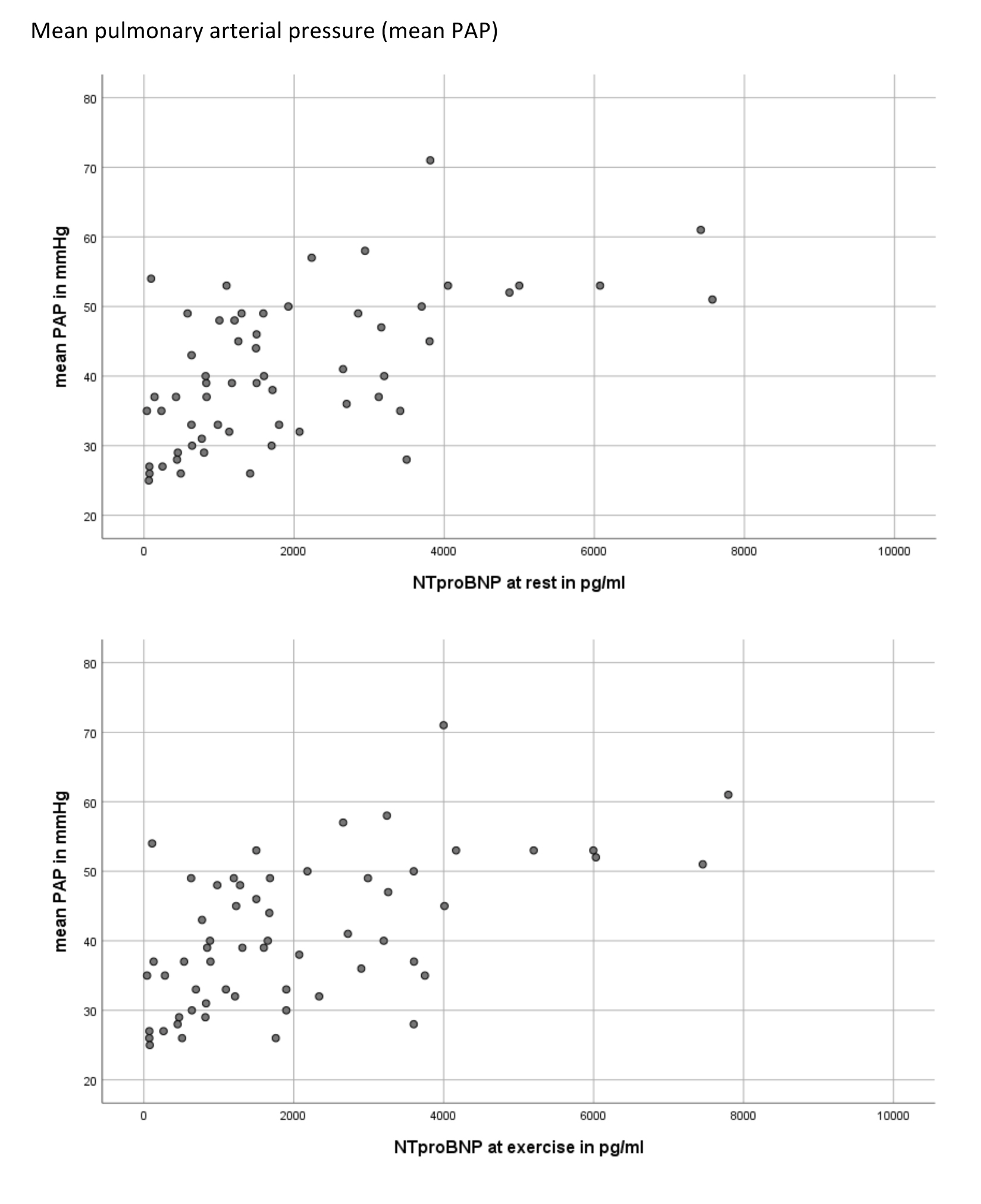

Supplement: Additional file 1: — Correlation plots of NT-proBNP at rest and at peak exercise and the ΔNT-proBNP with the 6MWT distance, mean PAP and the VO2/kg. (ZIP 540 kb) [file 12931_2017_712_MOESM1_ESM.zip › RERE-D-17-00298-R2-Figure Part 2.jpg]

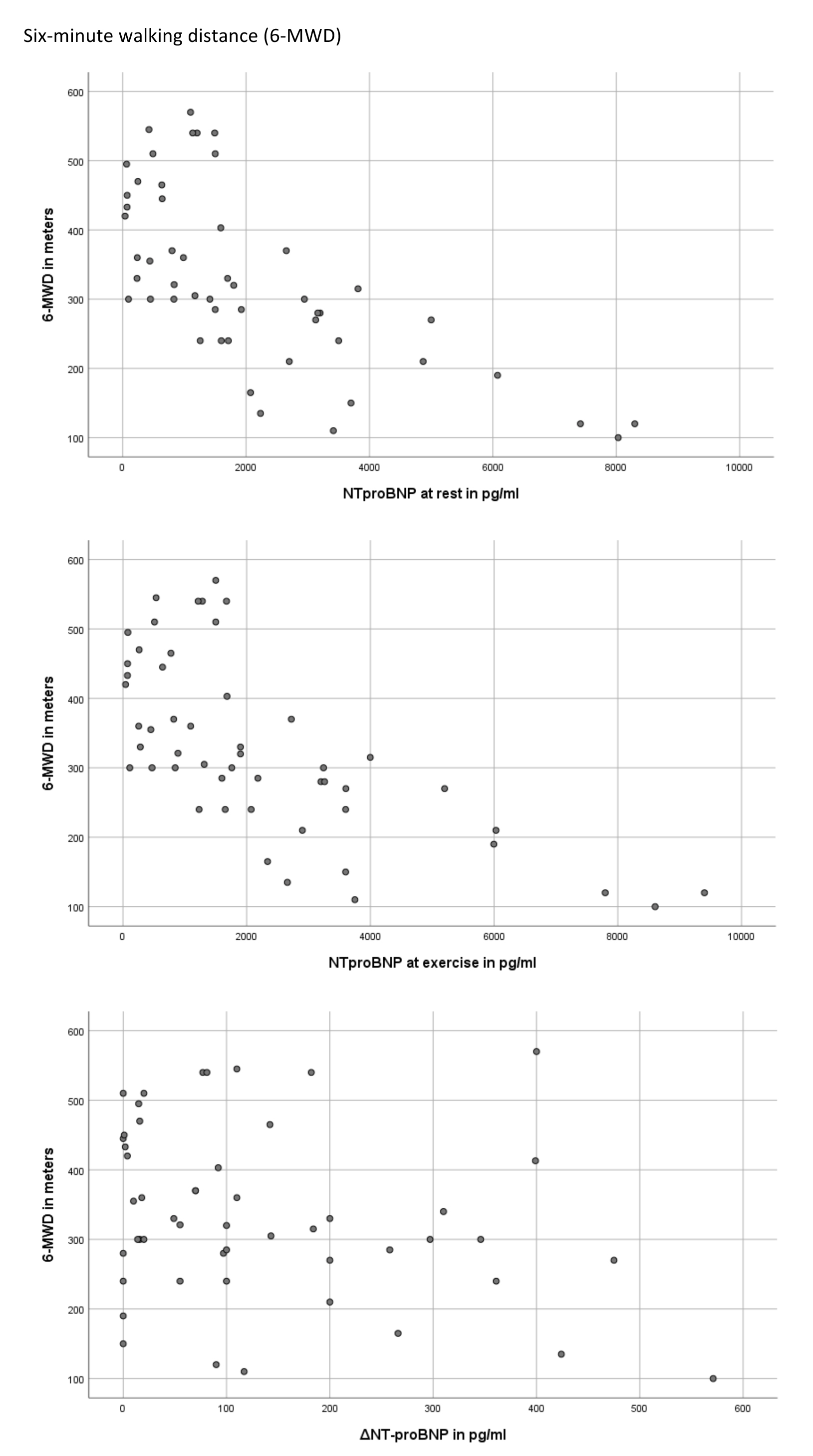

Supplement: Additional file 1: — Correlation plots of NT-proBNP at rest and at peak exercise and the ΔNT-proBNP with the 6MWT distance, mean PAP and the VO2/kg. (ZIP 540 kb) [file 12931_2017_712_MOESM1_ESM.zip › RERE-D-17-00298-R2-Figure Part 1.jpg]
